# Critical Roles of Lysophospholipid Receptors in Activation of Neuroglia and Their Neuroinflammatory Responses

**DOI:** 10.3390/ijms22157864

**Published:** 2021-07-23

**Authors:** Bhakta Prasad Gaire, Ji-Woong Choi

**Affiliations:** College of Pharmacy and Gachon Institute of Pharmaceutical Sciences, Gachon University, Yeonsu-gu, Incheon 21936, Korea; samarpanbp@gmail.com

**Keywords:** lysophospholipid receptors, S1P, LPA, microglia, astrocyte, neuroinflammation

## Abstract

Activation of microglia and/or astrocytes often releases proinflammatory molecules as critical pathogenic mediators that can promote neuroinflammation and secondary brain damages in diverse diseases of the central nervous system (CNS). Therefore, controlling the activation of glial cells and their neuroinflammatory responses has been considered as a potential therapeutic strategy for treating neuroinflammatory diseases. Recently, receptor-mediated lysophospholipid signaling, sphingosine 1-phosphate (S1P) receptor- and lysophosphatidic acid (LPA) receptor-mediated signaling in particular, has drawn scientific interest because of its critical roles in pathogenies of diverse neurological diseases such as neuropathic pain, systemic sclerosis, spinal cord injury, multiple sclerosis, cerebral ischemia, traumatic brain injury, hypoxia, hydrocephalus, and neuropsychiatric disorders. Activation of microglia and/or astrocytes is a common pathogenic event shared by most of these CNS disorders, indicating that lysophospholipid receptors could influence glial activation. In fact, many studies have reported that several S1P and LPA receptors can influence glial activation during the pathogenesis of cerebral ischemia and multiple sclerosis. This review aims to provide a comprehensive framework about the roles of S1P and LPA receptors in the activation of microglia and/or astrocytes and their neuroinflammatory responses in CNS diseases.

## 1. Introduction

Glial cells are non-neuronal central nervous system (CNS)-resident cells that support neurons for CNS homeostasis and normal neuronal functioning in a healthy condition [1]. Abundantly distributed in the CNS, glial cells are in continuous communication with neurons, immune cells, and blood vessels [2,3]. They are primarily divided into four phenotypes, namely, astrocytes, oligodendrocytes, microglia, and ependymal cells [1]. Ependymal cells are present at the lining of the ventricular system. Their roles during CNS injury remain unclear. Oligodendrocytes are myelin-producing cells that play important roles in myelin-degenerating diseases such as multiple sclerosis (MS), neuromyelitis optica, and idiopathic inflammatory demyelinating diseases [4]. Microglia and astrocytes are considered major glial cell types that are critical regulators of brain injury and recovery in diverse neuroinflammatory disorders. Microglia are innate immune cells of the CNS that play important roles in the host defense [5,6]. Astrocytes are in close proximity to neurons and blood vasculatures [7]. Both microglia and astrocytes are the most motile and active glial cells in the CNS. They can sense any changes in the CNS milieu through their processes. Therefore, microglia and astrocytes are the primary cells to be activated upon any hazardous stimuli [8,9]. Activation of microglia and astrocytes in acute CNS injuries is necessary for the host defense [10]. For instance, activated microglia are involved in brain cleaning system as they can remove dead cells and tissue debris [11], whereas activated astrocytes can prevent neurodegeneration through the formation of glial scar [12]. However, chronic activation of microglia and astrocytes are considered as detrimental because these activated cells can promote neuroinflammatory events and ultimately lead to neurodegeneration [13].

Bioactive lysophospholipids and their receptors (lysophospholipid receptors) are believed to be potential targets for drug design to treat various diseases, which ensures the possibility for an entirely new class of lipidomic-based therapeutic agents [14,15]. Originally, lysophospholipids were thought to be precursors and metabolites in the biosynthesis of membrane phospholipids [16,17,18]. Later, two of them, lysophosphatidic acid (LPA) and sphingosine 1-phosphate (S1P), were identified as important extracellular signaling molecules that could participate in various biological functions in organisms, including immunity, inflammation, muscle contraction, development, fibrosis, obesity, cancer, angiogenesis, cellular migration, and neurite extension [17,19,20,21]. Being extracellular signaling molecules, LPA and S1P can signal through binding to and activating at least 11 cognate G protein-coupled receptors (six LPA receptors, LPA_1_–LPA_6_ and five S1P receptors, S1P_1_–S1P_5_) and mediate a variety of biological functions throughout the body [22,23,24]. LPA and S1P are abundantly present in the CNS, where receptor-mediated LPA and S1P signaling are believed to play crucial roles in neurological disorders involving neuroinflammation, the major cause of neurodegeneration [25,26,27,28]. In fact, LPA and S1P receptors have emerged as novel and fascinating therapeutic targets for several inflammatory CNS diseases, including MS, neuropathic pain, spinal cord injury, cerebral ischemia, traumatic brain injury, hydrocephalus, fetal hypoxia, seizure, hearing loss, Sandhoff disease, and neuropsychiatric disorders [17,20]. In most of these CNS diseases, neuroinflammatory responses initiated by activated microglia and astrocytes are considered major neuroharmful events. In fact, previous studies have revealed that these neuroglial cells express most LPA and S1P receptors [29,30,31,32,33]. Therefore, LPA and S1P receptors can influence glial cell biology, including activation, proliferation, migration, etc. In addition, LPA and S1P receptors can regulate inflammatory responses of microglia and astrocytes, consequently signifying that these receptors are potential drug targets for treating various neuroinflammatory disorders. The aim of this review is to explore the roles of receptor-mediated LPA and S1P signaling in neuroinflammation through the regulation of responses of microglia and astrocytes.

## 2. S1P Receptors in Activation of Microglia and Their Neuroinflammatory Responses

A growing body of evidence has revealed the involvement of S1P receptors in microglial biology in diverse neuroinflammatory disorders. For example, it has been suggested that S1P might be involved in the migration and morphological alteration of microglia [34,35]. In cultured microglia, S1P can influence ATP release via volume-regulated anion channels [35]. The released ATP is associated with the migration of microglia [35]. S1P lyase-deficient microglia, in which amounts of S1P are found to be increased, show a significant decrease in ramification index of microglia (a morphological phenotype indicating microglial activation), along with increased ionized calcium-binding adapter molecule 1 (Iba1) reactivity [34], suggesting that S1P can promote microglial activation. In addition, mRNA and protein expression levels of proinflammatory mediators (such as tumor necrosis factor-α (TNF-α) and interleukin-6 (IL-6)) and mRNA expression levels of Toll-like receptor 4 (TLR4) are significantly increased in S1P lyase-deficient microglia [34], demonstrating that increased S1P levels are associated with microglial activation and their proinflammatory responses. In mice brains, microinjection of S1P into the corpus callosum can dramatically increase microglial activation, as evidenced by an increased number of Iba1-positive cells [27]. Such an S1P-induced microglial activation is closely associated with increased brain damage upon induction of transient middle cerebral artery occlusion (tMCAO), a type of focal cerebral ischemia [27]. Similarly, in microglia subjected to oxygen–glucose deprivation/re-oxygenation (OGD/R), a popular in vitro model of cerebral ischemia [36,37], S1P exposure can increase the production of IL-17A, which is associated with neuronal apoptosis [38]. Besides S1P itself, sphingosine kinase 1 (SphK1), a principal kinase responsible for S1P production in the brain [39], is also a key regulator of microglial activation and subsequent production of inflammatory mediators [40]. Protein expression levels of SphK1 are upregulated in lipopolysaccharide (LPS)-stimulated BV2 microglia cell line [40]. Suppression of SphK1 activity with either pharmacological or genetic tools can attenuate the upregulation of inflammatory mediators such as TNF-α, IL-1β, and inducible nitric oxide synthase (iNOS) [40], further indicating the role of S1P in neuroinflammatory activation of microglia.

The involvement of S1P receptors in microglial activation and their inflammatory responses has been further validated through experimental studies employing FTY720 (fingolimod, Gilenya^®^, Novartis, Switzerland), a drug to treat MS. FTY720 is a non-selective modulator of S1P receptors [41,42] with a pharmacological potential as a functional antagonist of S1P_1_ [43,44,45] and S1P_3_ [46]. It can decrease microglial activation in diverse CNS diseases, including Alzheimer’s disease (AD), Parkinson’s disease (PD), cerebral ischemia, and MS [47]. FTY720 can significantly attenuate mRNA and protein expression levels of proinflammatory cytokines such as TNF-α, IL-1β, and IL-6 in LPS-stimulated mouse primary microglia [48]. In addition, it can decrease lysophosphatidylcholine-induced production of nitric oxide (NO), TNF-α, and IL-1β from microglia [49]. In a kainic acid-induced neurodegenerative mouse model, FTY720 can significantly decrease the number of Iba1-positive cells in the brain and attenuate JNK phosphorylation in LPS-stimulated microglia [50]. In mice exposed to chronic unpredictable mild stress, FTY720 can decrease depressive-like behaviors by attenuating hippocampal NOD-like receptor pyrin domain-containing protein 3 (NLRP3) inflammasome activation [51], which is believed to occur in microglia because microglia are central regulators of NLRP3 inflammasome activation in the brain [52]. FTY720 decreases mRNA expression levels of iNOS and CD16, but increases mRNA expression levels of arginase 1 (Arg-1) and CD206, suggesting that FTY720 can promote M2 polarization of activated microglia (anti-inflammatory/neuroprotective phenotype) while attenuating M1 polarization (proinflammatory/neuro-harmful phenotype) [51]. FTY720 and SEW2871 (a selective agonist for S1P_1_ [53]) can significantly attenuate microglial activation in the substantia nigra of 1-methyl-4-phenyl-1,2,3,6-tetrahydropyridine (MPTP)-induced PD mouse [54], further indicating the role of S1P receptors in microglial biology. In an ischemic stroke-challenged brain, FTY720 can attenuate microglial activation and M1 polarization as evidenced by attenuated protein expression levels of Iba1, iNOS, and NLRP3 [55]. The effect of FTY720 on microglial polarization in cerebral ischemia is further confirmed in OGD/R-challenged microglia, in which FTY720 treatment decreases mRNA expression levels of M1 polarization markers such as CD86, cyclooxygenase-2 (COX-2), iNOS, IL-1β, IL-6, TNF-α, and interferon-γ (IFN-γ), whereas it increases mRNA expression levels of M2 polarization markers and growth factors such as transforming growth factor-β1 (TGF-β1), TGF-β2, TGF-β3, C-C motif chemokine ligand 2 (CCL2), granulocyte colony-stimulating factor (GCSF), granulocyte-macrophage colony-stimulating factor (GMCSF), and insulin-like growth factor-1α (IGF-1α) [55]. FTY720 can decrease mRNA expression levels of proinflammatory cytokines and chemokines such as IL-1α, IL-1β, TNF-α, IL-6, CCL2, CCL3, CCL4, and CCL9 in LPS-stimulated primary microglial culture [56]. In addition, FTY720 treatment can significantly decrease the number of activated microglia at the proximity of beta-amyloid (Aβ) plaque in AD-prone transgenic mice [57]. Similarly, following a hypoxic/ischemic insult in mice, FTY720 treatment can attenuate microglial activation and upregulation of proinflammatory cytokines such as TNF-α and IL-1β at protein and mRNA expression levels in the white matter of the brain [58]. FTY720 can also attenuate microglial activation in twitcher mice as numbers of Iba1-positive cells and ameboid microglia in the white matter of the cerebellum are decreased after treatment with FTY720 [59,60]. Findings of these in vitro and in vivo studies suggest that FTY720 can promote the anti-inflammatory phenotype and reduce the proinflammatory phenotype of microglia.

Besides FTY720, a few other S1P receptor modulators can also influence microglial activation, further strengthening the notion that receptor-mediated S1P signaling plays an important role in the activation of microglia and their inflammatory responses. In the culture of corticostriatal slices obtained from mice with experimental autoimmune encephalomyelitis (EAE) as a popular animal model of MS [61,62], ozanimod (RPC1063, Zeposia^®^, Bristol Myers Squibb), a recently developed drug to treat MS by targeting S1P_1_ and S1P_5_ [63], can attenuate M1 microglial activation, as evidenced by reduced mRNA expression levels of Iba1, iNOS, IL-1β, and TNF-α, while it induces M2 microglial activation, as evidenced by increased mRNA expression levels of Found in Inflammatory Zone 1 (FIZZ1) [63]. These ex vivo effects of ozanimod on M1 microglial activation were further validated in Th1 cytokines-stimulated BV2 microglia, in which ozanimod decreased mRNA expression levels of IL-6, Regulated upon Activation, Normal T Cell Expressed and Presumably Secreted (RANTES), and TNF-α [64]. Siponimod (BAF312, Mayzent^®^, Novartis) is also a drug used to treat MS by targeting S1P_1_ and S1P_5_ [65]. It can dramatically attenuate microglia activation in the brains of EAE mice [66]. In addition, RP-001, a selective agonist for S1P_1_ [67], can attenuate microglial activation in mice brains challenged with sub-arachnoid hemorrhage, as evidenced by a significant reduction in Iba1 immunoreactivity [68]. Besides cerebral ischemia and MS, receptor-mediated S1P signaling could also influence other neurodegenerative diseases. FTY720 administration can significantly reduce protein expression levels of proinflammatory mediators (COX-2 and TNF-α) in the hippocampus of Aβ-induced AD-like Wistar rat [69]. FTY720 can also dramatically attenuate microglial activation in 5xFAD AD-like mice [57,70]. In addition, it can significantly decrease the number of ameboid microglia in 5xFAD mice while there is no difference in the number of resting microglia upon FTY720 administration [57], further demonstrating that FTY720 can attenuate microglial activation in AD mice. Ameboid microglia are neurotoxic [71,72]. FTY720 can reduce neurotoxic microglia by attenuating inflammatory microglial activation in an AD mouse brain [57]. Similarly, in APP/PS1 mice, FTY720 administration can attenuate microglial activation, as evidenced by the suppressed Iba1 expression in the hippocampus and neocortex [73,74]. FTY720 can also attenuate microglial activation in experimental models of PD [75,76]. It can attenuate the increase in the number of Iba1-immunopositive cells in the substantia nigra and striatum at 21 days after mice are exposed to 6-hydroxydopamine (6-OHDA) [75]. These studies clearly reflect the involvement of S1P receptors in microglia-mediated neuroinflammatory events during CNS pathogenesis. These aforementioned independent studies clearly indicate that S1P itself and its receptor can influence microglial activation, possibly leading to inflammatory responses in diverse neurodegenerative diseases. In the following sections, we will discuss how each S1P receptor can affect the activation of microglia and their neuroinflammatory responses.

Regulatory roles of S1P_1_ in the activation of microglia and their neuroinflammatory responses have been well demonstrated in cerebral ischemia and MS. In cerebral ischemia, S1P_1_ plays a pathogenic role with a close link to neuroinflammation, mainly by influencing microglial activation [77,78]. Suppressing S1P_1_ activity with the administration of AUY954, one of its functional antagonists [43], can reduce proinflammatory responses and enhance anti-inflammatory responses of activated microglia in post-ischemic brains [78]. Experimental studies employing an S1P_1_ knockdown approach also support these findings as S1P_1_ knockdown can reduce mRNA expression levels of proinflammatory cytokines in LPS-stimulated cultured microglia [77]. In fact, suppressing S1P_1_ activity with either AUY954 administration or S1P_1_ knockdown can attenuate brain damages after tMCAO challenge [77]. Such neuroprotection by AUY954 is closely associated with attenuation of microglial activation, proliferation, and morphological transformation into ameboid cells in the brains of mice challenged with tMCAO [77]. Furthermore, S1P_1_ can regulate the activation of mitogen-activated protein kinases (MAPKs) and microglial NF-κB signaling pathways in post-ischemic brains [78]. Both MAPKs and NF-κB signaling pathways are associated with the regulation of neuroinflammation in cerebral ischemia [79,80,81]. In MS pathogenesis, modulation of S1P_1_, presumably inhibition, can lead to the attenuation of microglial activation [82,83,84]. FTY720 modulates microglial activation in the injured spinal cords of EAE mice, in which FTY720 administration dramatically attenuates mRNA upregulation of proinflammatory mediators, including C-X-C motif chemokine ligand 9 (CXCL9), CXCL11, CXCL13, CCL1, CCL2, CCL4, CCL5, CCL7, CCL17, Axl, FosB, Fos, and TNF-α, while it increases the upregulation of colony-stimulating factor 2 (CSF2), Chi3l3, IL-10, IGF-1, Rentla, and CD206 [84]. In the corpus callosum of cuprizone-administered mouse, in another model of MS with a feature of demyelination [85,86], FTY720 administration can dramatically decrease the number of Iba1-immunopositive cells and upregulate mRNA expression levels of proinflammatory cytokines and chemokines [83]. These effects of FTY720 might be mediated through downregulation of S1P_1_ in the brain because FTY720 administration completely abolishes cuprizone-induced protein expression levels of S1P_1_ in the brain [83]. Such possible involvement of S1P_1_ has been reaffirmed with CYM5442, another S1P_1_ selective modulator and a functional antagonist of S1P_1_ [82], indicating that S1P receptor modulation, possibly S1P_1_ modulation, could dramatically attenuate neuroinflammatory responses of microglia in MS.

S1P_2_ also plays a critical role in the activation of microglia and their inflammatory responses in post-ischemic brains [87]. JTE013, an antagonist for S1P_2_ [88,89], can dramatically attenuate microglial activation at day 1 and day 3 after ischemic challenge (tMCAO challenge) [87]. It can also attenuate microglial proliferation and mRNA upregulation of proinflammatory mediators in post-ischemic brains [87]. Importantly, JTE013 administration into tMCAO-challenged mice can lead to the attenuation of NF-κB signaling in activated microglia, suggesting that suppression of S1P_2_ activity can attenuate transcriptional activation of proinflammatory mediators in post-ischemic brains [87]. The inhibition of inflammatory microglial activation upon pharmacological inhibition of S1P_2_ in a post-ischemic brain could be the associated neuroprotective mechanism achieved by either JTE013 administration or genetic deletion of S1P_2_ against tMCAO challenge [87,90]. JTE013 can also decrease mRNA expression levels of proinflammatory cytokines in BV2 murine microglia upon LPS stimulation [87], suggesting that S1P_2_ is a critical regulator of microglial activation. However, how S1P_2_ regulates microglial activation in other CNS pathologies remains unclear.

Roles of S1P_3_ in the activation of microglia and their proinflammatory responses have also been reported in cerebral ischemia. Blockade of S1P_3_ with CAY10444, a specific S1P_3_ antagonist [91,92,93], can decrease the number of Iba1-positive cells, microglial proliferation, morphological transformation into neurotoxic ameboid shape, and inflammatory M1 polarization of microglia [94]. In addition, S1P_3_ can influence the activation of microglial NF-κB and the production of inflammatory cytokines in injured brains after an ischemic challenge [94]. In primary microglia, suppressing S1P_3_ activity with either CAY10444 or S1P_3_-specific shRNA lentivirus particles can dramatically attenuate mRNA upregulation of proinflammatory cytokines, suggesting that S1P_3_ is involved in the proinflammatory activation of microglia [94]. Unsurprisingly, S1P_3_ has been validated as a therapeutic target for drug development to treat cerebral ischemia because CAY10444 administration can attenuate brain damages such as brain infarction, neurological functional deficit, and neural cell death after tMCAO challenge [94]. However, the role of S1P_3_ in microglial activation-associated other CNS disorders is yet to be uncovered.

S1P_4_ is poorly expressed in the brain. Therefore, brain cells do not express S1P_4_ under normal conditions [95,96]. However, under disease conditions, expression levels of S1P_4_ in the brain are increased because infiltrated cells, particularly T cells, express S1P_4_ [95,96]. S1P_4_ is also expressed on neutrophils known to play important roles in neuroinflammatory responses under disease conditions [97,98]. S1P_4_ can regulate the migration and infiltration of neutrophils and their inflammatory responses through 5-lipoxygenase activity [97]. In injured brains after an ischemic challenge, mRNA expression levels of S1P_4_ are increased and peaked at 4 days after tMCAO challenge [99]. Similarly, in injured spinal cords of EAE mice, mRNA expression levels of S1P_4_ are upregulated [100]. These independent in vivo studies indicate that S1P_4_ plays a critical role in the pathogenesis of stroke and MS. However, whether S1P_4_ can directly regulate the activation of microglia and their neuroinflammatory responses requires further investigation.

S1P_5_ is moderately expressed on microglia [101]. Being a target of FTY720, S1P_5_ must have certain roles in microglial biology, including microglial activation and proinflammatory responses. In fact, S1P_5_ is associated with microglial activation because an S1P_5_ agonist can significantly increase the number of Iba1-positive cells in organotypic cerebellar slice cultures [102]. Upon stimulation with Th1 cytokines, ozanimod can significantly reduce mRNA expression levels of proinflammatory cytokines including IL-6, RANTES, and TNF-α in striatal slice cultures from EAE mice as well as in Th1-stimulated BV2 microglial cells [64]. In addition, ozanimod can decrease the number of Iba1/IL-1β-double immunopositive cells in corticostriatal slices from EAE mice [64], suggesting that it can inhibit inflammatory microglial activation. Considering that ozanimod can decrease mRNA expression levels of IL-1β and TNF-α while increasing FIZZ1 in Th1 cytokines-activated BV2 cells [64], ozanimod not only can inhibit M1 microglial phenotype but also can promote M2 microglial phenotype in EAE.

## 3. S1P Receptors in Activation of Astrocytes and Their Neuroinflammatory Responses

Receptor-mediated S1P signaling has been well characterized in astrogliosis (astrocyte activation) under different disease conditions. In the blood, platelets are considered the major source of S1P production [103,104]. In the CNS, astrocytes play such a role [105,106]. In fact, cerebellar astrocytes can release S1P [105,106] to influence the proliferation of astrocytes by activating ERK1/2 signaling [106]. In spinal cord injuries, reactive astrocytes mainly found in injured areas can produce S1P, indicating that astrocytes are major sources of S1P in an injured spinal cord [107]. In addition to the role of cells producing S1P, astrocytes themselves can be activated by S1P. In addition, S1P can induce migration of astrocytes and promote the secretion of CXCL1, a chemokine, from reactive astrocytes [108], suggesting that S1P can regulate the biology of astrocytes and their inflammatory responses. Therefore, S1P-influenced inflammatory astrogliosis can trigger the pathogenesis of diverse neuroinflammatory diseases. Indeed, it has been reported that S1P microinjection can result in a significant increase in astrogliosis in the corpus callosum of either normal or stroke-challenged mice [27]. The observed astrogliosis has been thought to occur possibly via S1P_1_ activation because the administration of FTY720 with the highest binding affinity to S1P_1_ among S1P receptors [109,110] can decrease the number of reactive astrocytes in the corpus callosum of normal or stroke-challenged mice [27]. FTY720 administration can also reduce mRNA expression levels of proinflammatory mediators (IL-6, IFN-γ, IL-1β, TNF-α, IL-12A, IL-23A, CXCL10, CCL2, CCL20, and NOS2) in astrocytes isolated from EAE mice [84]. Conversely, mRNA expression levels of anti-inflammatory mediators such as CXCL12 and IL-33 are upregulated after FTY720 administration [84]. In addition, microinjection of exogenous S1P into the striatum of a mouse brain can increase astrogliosis [111], suggesting that S1P can induce neuroinflammation by promoting astrogliosis. Most S1P receptors (S1P_1_, S1P_2_, S1P_3_, and S1P_5_) are expressed on astrocytes [95,105,112]. They can influence diverse biological events of astrocytes, such as proliferation, migration, communication between astrocytes and the blood–brain barrier gap junction, and production of growth factors [95]. Among S1P receptors expressed on astrocytes, S1P_1_ is the highest expressed receptor type, followed by S1P_3_ [112,113]. Although the basal gene expression level of S1P_5_ is undetectable, its expression has been found to be increased in cultured astrocytes upon growth factor supplementation for 4–6 days [112,113]. Growth factor supplement (containing epidermal growth factor, basic fibroblast growth factor, insulin, biotin, human transferrin, and hydrocortisone) can dramatically increase mRNA expression levels of S1P_5_ in cultured astrocytes [113]. Among S1P receptors, S1P_1_ and S1P_3_ can promote inflammatory activation of astrocytes, which has been well characterized in different disease conditions [13,114,115].

The neuroinflammatory roles of S1P_1_ in astrocytes have been thoroughly studied in MS [84,116]. Protein expression levels of S1P_1_ and S1P_3_ are significantly increased in active MS lesions [117]. Upregulated S1P_1_ and S1P_3_ are mainly detected in reactive astrocytes [117], indicating that activation of these receptors on astrocytes may trigger inflammatory responses, thereby playing a role in the pathogenesis of MS. In human autopsy brain samples from MS patients, S1P_1_ has been found to be mainly upregulated in astrocytes and blood vessels, but not in myelin sheath or microglia/macrophages [118], suggesting that S1P_1_ on astrocytes is a critical pathogenic player in MS. In fact, neuroprotective effects of FTY720 in MS are mainly associated with modulation of astrocytic S1P_1_ activity [43,82,119,120]. In EAE, selective deletion of S1P_1_ from astrocytes, but not from neurons, can improve disease severity, demonstrating that astrocytic S1P_1_ is responsible for the pathogenesis of MS [43]. S1P_1_ deletion from astrocytes can also reduce protein expression levels of proinflammatory cytokines [43]. FTY720 can reduce migration and proliferation of S1P-stimulated cultured astrocytes mainly by downregulating S1P_1_ [121]. These effects of FTY720 can ameliorate spinal cord injury-induced functional deficits in rats [121]. In addition, FTY720 can influence astrocytes proliferation and reduce the secretion of inflammatory chemokines, astrogliosis, and neuroinflammation (reviewed well in [122]), all of which are associated with MS pathogenesis [123,124,125]. Furthermore, FTY720 treatment can decrease mRNA expression levels of proinflammatory mediators such as CCL2, NOS2, CSF2, IL-6, and TNF-α, while increasing mRNA expression levels of anti-inflammatory cytokines such as IL-10 in LPS-activated astrocytes culture [84], indicating its potent anti-inflammatory efficacy in activated astrocytes. In EAE mice, FTY720 can attenuate the upregulation of IL-1 receptor (IL-1R), S1P_1_, S1P_3_, iNOS, and nitrotyrosine in astrocytes [126]. Neuroprotective effects of FTY720 in EAE are also mediated through the blockade of NO production and nuclear translocation of NF-κB in astrocytes [126]. These effects of FTY720 on inflammatory astrogliosis and MS pathogenesis might be due to its ability to suppress S1P_1_ activity [127]. Similarly, CYM-5442, a modulator of S1P_1_, can attenuate the severity of EAE and reduce amounts of proinflammatory mediators such as monocyte chemoattractant protein 1 (MCP-1), IL-17, IL-10, and IL-1β in blood plasma [82]. These effects of CYM-5442 are comparable to those of FTY720 [82]. S1P_1_ is upregulated on neurons and astrocytes during EAE. Such upregulation is significantly attenuated by CYM-5442 treatment, indicating that downregulating S1P_1_ activity in astrocytes can reduce neuroinflammation and the severity of MS pathogenesis, at least in part [82]. Matrine, a natural alkaloid component extracted from *Sophorae flavescens*, can also attenuate astrogliosis in the brains of EAE mice [128]. This effect is mediated by lowering S1P amounts and suppressing SphK1/2 activity [128]. Matrine can also attenuate S1P_1_ upregulation in astrocytes in the brains of EAE mice [128], suggesting that the neuroprotective effects of Matrine in EAE mice are mediated through downregulation of S1P_1_ in astrocytes, which is reaffirmed in cultured astrocytes stimulated with IFN-γ [128]. S1P_1_ activation can also trigger ERK1/2 phosphorylation in mixed glial culture and astrocytes [129]. Since ERK1/2 signaling could contribute to MAPK-mediated inflammatory responses [130,131,132], S1P_1_ activation can promote such inflammatory responses. All these findings clearly indicate that astrocytic S1P_1_ is the major S1P receptor subtype responsible for neuroinflammation and disease pathogenesis of MS by modulating astrogliosis.

Roles of receptor-mediated S1P signaling, possibly through S1P_1_, in astrocytes have been indicated in other CNS diseases as well. Siponimod can block inflammatory activation of astrocytes as it can reduce nuclear translocation of NF-κB in IL-1β- or IL-17-stimulated astrocytes [133]. On the other hand, siponimod significantly increases expression levels of nuclear factor erythroid 2-related factor 2 (Nrf2), a potent antioxidant regulator [134]. These independent studies suggest its dual effects on astrocytes. Siponimod can also attenuate cell death of spinal neurons exposed to conditioned medium of S1P- or IL-1-treated astrocytes and induce S1P_1_ internalization [135]. Although whether siponimod can block S1P_1_ recycling is not determined in that study [135], siponimod might reduce S1P-induced neurodegeneration via a functional antagonism of S1P_1_ in astrocytes. FTY720 can enhance the recovery after contusion-induced spinal cord injury in mice by attenuating the accumulation of reactive astrocytes in injured areas [136], which could be mediated by suppressing S1P_1_ activity because FTY720 has been proven to have a functional antagonistic effect on S1P_1_ [43,44,45]. Similarly, in APP/PS1 mice, FTY720 administration can attenuate astrocyte activation as evidenced by attenuated glial fibrillary acidic protein (GFAP) expression in the hippocampus and neocortex [73,74]. In addition, it can increase astrocytic phagocytosis of Aβ, suggesting that it not only attenuates neuroinflammatory activation of astrocytes but also contributes to the clearance of Aβ plaque in AD pathogenesis [74]. FTY720 can also attenuate astrocyte activation in experimental models of PD [74,75]. It can decrease the number of GFAP-immunopositive cells in the substantia nigra and striatum at 21 days after exposing mice to 6-hydroxydopamine (6-OHDA) [75]. These studies indicate that FTY720 can exert its neuroprotective effects by attenuating astrocyte activation in different CNS pathogenesis. Such anti-neuroinflammatory activities of FTY720 in astrocytes might be mediated by downregulation of S1P_1_ activity as FTY720 has a functional antagonistic effect on S1P_1_ [43,44,45].

S1P can trigger the upregulation of mRNA and protein expression levels of COX-2 in astrocytes through the Gα12/13-Rho pathway [137]. This S1P-triggered COX-2 upregulation is mediated through S1P_3_ activation because such upregulation is attenuated upon S1P_3_ knockdown or its pharmacological inhibition [137]. Since COX-2 is considered a proinflammatory mediator in diverse disease conditions [138], suppressing S1P_3_ activity could lead to attenuation of COX-2-mediated inflammatory responses. S1P_3_-mediated inflammatory responses in astrocytes have been reported by other independent studies as well. When murine or rat astrocytes are stimulated with TNF-α or LPS, mRNA expression levels of S1P_3_ are dramatically increased [139]. In addition, upon LPS stimulation, protein expression levels of S1P_3_ are increased in rat primary astrocytes [108]. Similarly, in cultured human astrocytes, mRNA expression levels of S1P_3_ are significantly increased upon TNF-α stimulation [117]. These independent studies indicate that astrocytic S1P_3_ can regulate inflammatory responses. Indeed, TNF-α-stimulated astrocytes can secrete MCP-1, an inflammatory mediator [117] involved in the pathogenesis of diverse neuroinflammatory diseases [140]. Moreover, astrocytic S1P_3_ has been reported to participate in the pathogenesis of several neuroinflammatory diseases. In MS lesions, S1P_3_ has been found to be dramatically upregulated in reactive astrocytes [108]. A recent study has revealed that S1P_3_ can regulate gene expression of inflammatory mediators in astrocytes through RhoA signaling pathways [137]. In a post-ischemic brain, pharmacological inhibition of S1P_3_ activity through administration of CAY10444 can attenuate astrogliosis, as evidenced by reduced GFAP immunoreactivity and astrocyte proliferation [94]. Particularly, at 3 days after the ischemic challenge, the number of reactive astrocytes is increased in injured brains, which is then reduced upon CAY10444 administration [94]. These results from a study using an in vivo cerebral ischemia model suggest that S1P_3_ can promote reactive astrogliosis in the post-ischemic brain. Similarly, in a mouse model of Sandhoff disease mainly characterized by reactive astrogliosis [141], genetic deletion of S1P_3_ can improve disease symptoms and attenuate astrogliosis [142]. In brain cancer, S1P_3_ is also upregulated in highly permeable metastases, mainly in astrocytes, where S1P_3_ influences the infiltration of peripheral immune cells and the production of inflammatory cytokines [143]. When astrocytic S1P_3_ activity is attenuated through its genetic knockdown, most inflammatory mediators (CCL2, CXCL1, CXCL10, intercellular adhesion molecule-1 (ICAM-1), IL-6, and IL-8) are downregulated at mRNA expression levels [143], reaffirming the neuroinflammatory roles of astrocytic S1P_3_. These studies clearly reflect the roles of S1P_3_ in reactive astrocytes and their neuroinflammatory responses during diverse pathological conditions. Mechanistically, neuroinflammatory roles of astrocytic S1P_3_ can be mediated through increased intracellular calcium signaling [91]. S1P-induced increase in intracellular calcium concentration is reduced upon pharmacological inhibition of S1P_3_ in rat primary cortical astrocytes, suggesting that S1P-influenced intracellular calcium stress is mediated by S1P_3_. As intracellular calcium overload is associated with diverse cellular responses, including neuroinflammation [144,145], suppressing S1P_3_ activity can reduce astrocyte-dependent neuroinflammatory events. Indeed, S1P-stimulated CXCL1 chemokine production from cultured astrocytes is attenuated upon pharmacological inhibition of S1P_3_ [91], further supporting the neuroinflammatory roles of S1P_3_ in astrocytes.

In a normal brain, S1P_4_ expression is negligible, whereas it is upregulated in an injured brain after an ischemic challenge [99]. However, how neuroglial S1P_4_ influences disease pathogenesis has not been reported yet. S1P_5_ is also highly expressed on astrocytes and oligodendrocytes of active MS lesions as much as S1P_1_ [118], indicating that S1P_5_ might also modulate neuroinflammatory responses of astrocytes. A previous study has revealed that siponimod can inhibit NF-κB activation in IL-1β-stimulated astrocytes [133], indicating possible roles of S1P_5_ in the activation of astrocytes and subsequent inflammatory responses. However, whether astrocytic S1P_5_ can influence neuroinflammatory events remains unclear. Instead, in oligodendrocytes, a main locus of S1P_5_ in the brain, S1P_5_ is known to have roles in regulating biological functions, including maturation, differentiation from neural stem cells to oligodendrocytes, and survival [146,147]. Further detailed studies about the roles of S1P_4_ and S1P_5_ in activation of astrocytes and their neuroinflammatory responses are needed.

Table 1 presents the biological roles of S1P receptors in the activation of neuroglia and their neuroinflammatory responses.

Although we discussed the role of receptor-mediated S1P signaling in the activation of microglia and/or astrocytes and their neuroinflammatory responses, there could be S1P-mediated glial crosstalk, resulting in neuroinflammatory responses. The proinflammatory mediators secreted by S1P-activated astrocytes or microglia might be able to further activate each other and prolong glial activation cascades. In fact, S1P signaling in both microglia and astrocytes has been reported to promote neuronal apoptosis [148], indicating a possible influence of S1P on neuron–glial crosstalk. However, whether S1P signaling is involved in the crosstalk between microglia and astrocytes remains unknown, which would be of interest for future studies.

## 4. LPA Receptors in Activation of Microglia and Their Neuroinflammatory Responses

LPA plays significant roles in microglial biology, such as motility, cytoskeletal architecture, membrane ruffling, glycolysis, brain-derived neurotrophic factor (BDNF) production, and ATP release [149,150]. Recent reports have suggested that LPA is one of the critical mediators of microglial activation [28,151,152], further indicating that LPA and its receptor-mediated signaling might have critical roles in the pathogenies of diverse CNS disorders. LPA exposure can increase the metabolic activities of cultured microglia [29]. Especially, when microglia are exposed to LPA, their morphology is changed to a round shape with shorter and more thickened processes than resting microglia [149]. These morphological changes of microglia are characteristic features of activated ameboid microglia playing important roles in the pathogenesis of diverse CNS disorders [71,72]. In addition to functions demonstrated in vitro, LPA can trigger microglial activation in vivo. In neuropathic pain, intrathecal injection of LPA can induce microglial MAPK phosphorylation and upregulation of genes involved in microglial activation [29,149,153]. Minocycline, a specific inhibitor of microglial activation, can decrease LPA-induced mechanical allodynia and thermal hyperalgesia [154]. These effects of minocycline are also observed in sciatic inflammatory neuropathy [155]. These two independent studies clearly suggest that LPA-mediated neuropathic pain is caused by microglial activation, at least in part. In addition, intrathecal LPA injection not only can increase the number of activated microglia in the dorsal horn of the spinal cord but also can skew ramified microglia towards more toxic ameboid microglia [154]. Such microglial activation and morphological transformation by intrathecal LPA injection are associated with p38 MAPK phosphorylation [154]. In fact, it has been well demonstrated that LPA-mediated microglial activation can lead to the development of neuropathic pain at an early stage and that LPA_1_ plays a critical role in this process [156,157].

Among LPA receptors, LPA_1_ is expressed at the highest level in the brain and spinal cord [28,158]. While LPA_1_ is well expressed on primary microglia, it is undetectable in immortalized cell lines, for example, murine BV2 microglial cells [29,149,152,158,159]. It is clear that LPA_1_ is associated with the activation of microglia and their inflammatory responses in diverse neuroinflammatory diseases. In post-ischemic brains, LPA_1_ is associated with microglial activation, proliferation, and inflammatory responses [160,161,162]. Suppressing LPA_1_ activity with LPA_1_-specific shRNA lentivirus or AM152 (a specific antagonist for LPA_1_, also known as BMS-986020 [163,164,165]) can decrease the number of Iba1-positive microglia in the brains of mice challenged with tMCAO [160,162]. It can also reduce the soma sizes of microglia and attenuate microglial proliferation and morphological transformation from ramified microglia into ameboid cells in injured brains after tMCAO challenge [160,162]. These data indicate that LPA_1_ is associated with microglial activation in post-ischemic brains. Importantly, suppressing LPA_1_ activity can dramatically attenuate microglial NF-κB expression in post-ischemic brains and decrease mRNA expression levels of proinflammatory cytokines in mouse primary microglia exposed to LPS [162]. These findings clearly reflect that LPA_1_ can influence the inflammatory activation of microglia. A recent report has also demonstrated that LPA_1_ is upregulated in the brains of mice challenged with intracerebral hemorrhage (ICH) and that such upregulation is mainly observed in microglia [166]. Suppressing LPA_1_ activity with AM966 (another specific antagonist for LPA_1_ [167]) not only can reverse microglial activation but can also improve long-term neurobehavioral functions following ICH [166], indicating that microglial LPA_1_ is critically involved in the pathogenesis of ICH as in that of cerebral ischemia [160,161,162]. In addition, in a mouse model of spinal cord injury caused by an intraspinal injection of LPA, microglial activation and demyelination are significantly increased [28]. Either LPA_1_ knockout or pharmacological inhibition of LPA_1_ with AM095, an LPA_1_-selective antagonist [168], can attenuate LPA-induced demyelination in the spinal cord and promote cell survival when oligodendrocytes are exposed to microglial conditioned medium treated with LPA, indicating that LPA_1_ is a critical mediator of LPA-induced microglial activation [28]. Microglial activation is a hallmark pathogenic event in neuropathic pain, in which LPA/LPA_1_ signaling is considered as a major factor in the pathogenesis of neuropathic pain [156,169,170]. LPA_1_ is also associated with microglial activation in an LPS-induced sepsis animal model [158]. In septic brains, LPA_1_ is associated with activation of microglia and their TNF-α production, which is recapitulated in LPS-stimulated rat primary microglia [158]. Suppressing LPA_1_ activity with LPA_1_-specific shRNA lentivirus can decrease the number of activated microglia and attenuate their proliferation in septic brains [158]. Similarly, in rat primary microglia, LPA_1_ knockdown with its specific siRNA can attenuate mRNA upregulation and protein release of TNF-α after LPS stimulation [158]. In SH-SY5Y cells co-cultured with BV2 microglia, saikosaponin-d, a natural triterpenoid saponin that acts as an inhibitor of LPA_1_ [171], can suppress neuronal LPA_1_ and MAP2 expression and reduce neuronal apoptosis as evidenced by increased protein expression levels of Bcl-2 with decreased Bax expression [171], revealing that microglial LPA_1_ is responsible for neuronal apoptosis. Saikosaponin-d not only can reduce microglial activation in the hippocampus of LPS-induced septic mouse, but also can attenuate proinflammatory cytokines production from LPS-stimulated primary microglia [172]. These aforementioned studies clearly suggest that LPA_1_ is a critical regulator of microglial activation, thus contributing to the pathogenesis of diverse CNS diseases.

LPA_2_ is expressed on microglia of the spinal cord, cultured primary microglia, BV2 murine microglia, and C13NJ microglia [149,152,173]. Microglial LPA_2_ is responsible for oligodendrocyte cell death, possibly by enhancing the production of purines and subsequent activation of P2X7 signaling [173]. Conditioned medium obtained from LPA-stimulated microglia can decrease the number of myelin basic protein (MBP)-positive cells and induce cell death of oligodendrocytes, suggesting that microglial LPA_2_ is responsible for this death of oligodendrocytes [173]. In fact, such regulatory roles of LPA_2_ are linked to the pathogenesis of spinal cord injury [173].

LPA_3_ is abundantly expressed on microglia [29]. Its expression is further increased in activated microglia [101]. Interestingly, microglial LPA_3_ is associated with LPA production [154,156]. The LPA/LPA_3_ signaling axis can influence the pathogenesis of neuropathic pain [154,156]. In addition, LPA_3_ can trigger the production of IL-1β from microglia, resulting in inflammatory cascades [154,170]. LPA_3_ is also associated with increased ERK1/2 phosphorylation in LPA-stimulated microglia [149]. Considering that ERK1/2 activation can trigger the production of inflammatory mediators [130,131,132], LPA_3_-regulated ERK1/2 activation might be an underlying event that promotes neuroinflammatory responses in microglia. In addition, LPA-induced membrane ruffling of activated microglia can be mediated through LPA_3_ [150] because it is attenuated by DGPP [150], a potent LPA_3_ antagonist [174]. Such findings [150] suggest that LPA_3_ is a critical modulator of microglial activation, indirectly supporting the previously reported roles of LPA_3_ in microglial neuroinflammatory responses [154,170]. In addition, Ki16425, a dual inhibitor of LPA_1_ and LPA_3_ [175], can attenuate LPA-induced ROS production in EOC mouse microglial cells, indicating that LPA_3_ can influence microglial oxidative stress [176]. Collectively, these studies indicate that LPA_3_ is associated with inflammatory microglial activation and oxidative stress under pathological conditions.

LPA_4_ is moderately expressed in a normal mouse brain, primary microglia, and BV2 microglia [158], suggesting its certain role in microglial biology. However, its role in microglial biology and related disease pathogenies has not been reported yet.

LPA_5_ is well expressed on microglia [177]. The association of LPA_5_ with microglial activation has been validated in both in vitro microglial culture and in vivo animal models. In LPA-stimulated primary microglia or BV2 microglial cell line, TCLPA5, a selective antagonist for LPA_5_ [178], can attenuate the upregulation of M1-related markers (such as iNOS, COX-2, CD40, and CD86) and proinflammatory cytokines (such as IL-1β and IL-6) [152]. In BV2 microglial cells, LPA_5_ knockdown with its specific siRNA can attenuate the production of proinflammatory cytokines (TNF-α, IL-1β, and IL-6) [179]. In addition, other LPA_5_ antagonists, such as AS2717638 and compound 3, can attenuate LPA-induced production of proinflammatory cytokines (TNF-α, IL-1β, and IL-6) and chemokines (CXCL10, CXCL2, and CCL5), all of which are markers of M1 microglia [180], indicating that the LPA/LPA_5_ signaling axis is important for the proinflammatory M1 activation of microglia. The LPA/LPA_5_ signaling axis is also associated with the activation of microglial MAPK signaling pathways, which subsequently promotes the activation of NF-κB and STAT1/3 signaling pathways in LPA-stimulated microglial cultures [181]. Importantly, LPA_5_ activation can increase the sizes of microglia and promote morphological transformation into ameboid-shaped microglia [181], further suggesting that LPA_5_ can promote inflammatory microglial activation in cultured microglia. This notion is also supported by studies employing an in vivo stroke model. In fact, LPA_5_ has been demonstrated to be a novel pathogenic factor for both acute and chronic ischemic injuries in mice subjected to tMCAO challenge because TCLPA5 not only can attenuate acute brain damage but also exert long-term neuroprotection in these mice [179,182]. Such a pathogenic role of LPA_5_ is closely associated with microglial activation in injured brains [179]. LPA_5_ can influence microglial activation and proliferation in post-ischemic brains [179]. The pharmacological inhibition of LPA_5_ with TCLPA5 can decrease the number of activated microglia in peri-ischemic and ischemic core regions and attenuate microglial proliferation in the penumbra region after tMCAO challenge [179]. TCLPA5 administration can also attenuate the morphological transformation of activated microglia from ramified into ameboid shape in post-ischemic brains [179]. In addition, it can attenuate the upregulation of M1-relevant proinflammatory markers (TNF-α, IL-1β, and IL-6) and microglial NF-κB activation in injured brains after tMCAO challenge [179]. The latter indicates that the observed LPA_5_-driven upregulation of M1-like proinflammatory cytokines may occur in microglia. This notion has been reaffirmed in cultured microglia in vitro [179,180]. Interestingly, LPA_5_ is upregulated in post-ischemic brains. Such upregulation is observed mostly in Iba1-positive cells (activated microglia) [179], indicating that LPA_5_ is the critical regulator of microglial activation in post-ischemic brains.

LPA_6_ is also highly expressed on mouse primary microglia and BV2 murine microglia [158]. However, its role in neuroinflammatory disease conditions remains unclear.

## 5. LPA Receptors in Activation of Astrocytes and Their Neuroinflammatory Responses

Receptor-mediated LPA signaling can also influence the activation of astrocytes in different CNS disease conditions. Autotaxin, a key enzyme of LPA synthesis [183], can promote adhesion of astrocytes and glioma invasion during glioblastoma multiforme pathogenesis [184], indicating that LPA can regulate astrocyte biology. LPA can induce the formation of stress fibers and focal adhesions [185] and increase intracellular calcium concentration in astrocytes, [186], all of which can stimulate astrocytic proliferation and migration [187,188,189,190] that are featured biological events of reactive astrocytes [190,191,192].

In a normal brain, LPA_1_ is highly expressed on astrocytes [113,166,193,194], indicating that LPA_1_ can influence astrocyte activation. A few recent studies have demonstrated such a role. In cultured astrocytes that express LPA_1_ abundantly, LPA can induce cell proliferation via LPA_1_ [195]. LPA can also promote the migration of LPS- or IL-1β-stimulated rat primary astrocytes mediated through LPA_1_ [196]. In C6 glioma cells, an immortalized astrocyte cell line, inhibition of LPA_1_ activity by Ki16425 (an LPA_1/3_ antagonist) or AM966 (an LPA_1_ antagonist) can attenuate LPA-triggered ERK1/2 activation, which is reaffirmed by LPA_1_ knockdown with its specific siRNA [197]. Inactivation of ERK1/2 by suppressing LPA_1_ activity could lead to attenuated proinflammatory responses in diverse CNS diseases such as cerebral ischemia [161,162]. In fact, inhibiting LPA_1_ signaling through its specific antagonist, AM095, can attenuate the activation and proliferation of astrocytes in post-ischemic brains, which might ultimately contribute to neuroprotection [162]. Besides cerebral ischemia, LPA_1_ may have certain roles in the activation of astrocytes and their neuroinflammatory responses in other disease types. As discussed earlier, either AM966 administration or genetic deletion of LPA_1_ can attenuate brain damages and neuroinflammatory responses in ICH-challenged mice [166]. In the injured brains of these mice, LPA_1_ is upregulated on reactive astrocytes, activated microglia, and neurons [166]. However, whether LPA_1_ could regulate astrocyte activation and whether the observed neuroinflammation is dependent on any of those cell types in ICH-challenged mice remains unknown. In a neuropathic pain model by a partial sciatic nerve ligation, LPA_1_ has been indicated as a possible mediator for the activation of astrocytes and their production of chemokine (CXCL1) in injured spinal dorsal horns, leading to the aggravation of pathogenesis because LPA_1/3_ antagonist (Ki16425) can attenuate CXCL1 upregulation in activated astrocytes [198]. Although LPA_1_ can influence a variety of functions of astrocytes [195,196], many of its roles in astrocyte-dependent neuroinflammation remain unclear.

LPA_2_ is also highly expressed on astrocytes [193], indicating that LPA_2_ might also play a critical role in neuroinflammatory responses. Similarly, other LPA receptors, including LPA_3_, LPA_4_, and LPA_6_, are also expressed on astrocytes [195,199], suggesting that these receptors might have certain roles in the neuroinflammatory responses of astrocytes. However, the roles of these LPA receptor subtypes in astrocyte activation and their neuroinflammatory responses have not been reported yet.

Table 2 presents the biological roles of LPA receptors in the activation of neuroglia and their neuroinflammatory responses.

## 6. Conclusions

**Figure 1:**
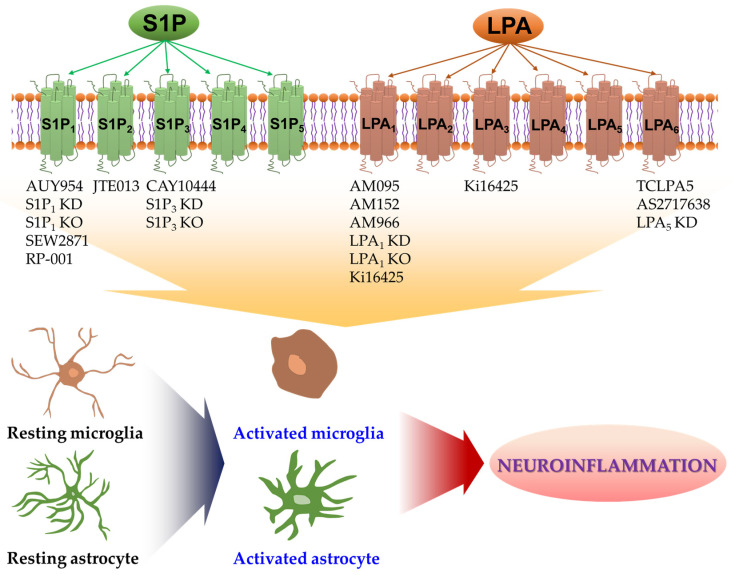
Either pharmacological or genetic approaches have been employed to identify roles of S1P and LPA receptors in glial activation under neuroinflammatory conditions. Receptor-mediated S1P and LPA signaling can promote the activation of microglia and astrocytes in various CNS pathologies. Activated microglia or activated astrocytes can secrete diverse proinflammatory mediators, including cytokines and chemokines, all of which can trigger neuroinflammatory events and contribute to CNS pathogenesis. Pharmacological or genetic tools listed below each receptor subtype can suppress the activity of the respective receptor. They have been reported to attenuate neuroinflammatory responses of activated microglia or activated astrocytes. Up to date, S1P_1_, S1P_2_, S1P_3_, S1P_5_, LPA_1_, and LPA_5_ have been identified as the receptors involved in such events. See text for details and references. KD: knockdown; KO: knockout.

Most LPA and S1P receptors can modulate neuroinflammatory responses by influencing the activation of microglia and astrocytes. S1P and LPA receptors are expressed on both cell types, and most of these expressed receptors are upregulated during CNS pathogenesis. Table 1 and Table 2 present the biological roles of S1P and LPA receptors in glial activation and their neuroinflammatory responses. Figure 1 schematically illustrates the neuroinflammatory responses of S1P and LPA receptors. Up to date, it has been reported that S1P_1_, S1P_2_, S1P_3_, S1P_5_, LPA_1_, and LPA_5_ can promote the activation of microglia and/or astrocytes and their neuroinflammatory responses. However, the roles of other receptors such as S1P_4_, LPA_2_, LPA_4_, and LPA_6_ remain unclear, possibly due to the lack of pharmacological modulators for these receptor subtypes. Therefore, future studies should focus on the development of receptor-specific modulators and the use of genetic tools for verifying the roles of specific receptor subtypes in the activation of microglia and/or astrocytes. Since the roles of LPA and S1P receptors in glial activation have been mainly studied in cerebral ischemia and MS, future studies should also focus on whether these receptors can influence the activation of microglia and/or astrocytes in other CNS diseases such as PD, AD, and Huntington’s disease. Moreover, developing new modulators of S1P and LPA receptor subtypes could be an appealing therapeutic option to manage CNS diseases featured by the activation of microglia and astrocytes and their inflammatory responses.

## Figures and Tables

**Table 1:** S1P receptors in activation of neuroglia and their neuroinflammatory responses. N/A: not available.

Receptors	Microglia	Astrocytes
S1P**_1_**	- AUY954 or S1P_1_ knockdown can attenuate microglial activation, morphological transformation into ameboid cells, and proliferation in cerebral ischemia [77]. - AUY954 can decrease mRNA expression levels of proinflammatory cytokines and attenuate microglial NF-κB activation in cerebral ischemia [78]. - AUY954 can reduce proinflammatory responses and enhance anti-inflammatory responses of microglia in cerebral ischemia [78]. - S1P_1_ knockdown can decrease mRNA expression levels of proinflammatory cytokines in LPS-stimulated primary microglia [77]. - SEW2871 can attenuate microglial activation in the substantia nigra in PD [54]. - RP-001 can attenuate microglial activation in sub-arachnoid hemorrhage [68].	- S1P_1_ deletion from astrocytes can reduce protein expression levels of proinflammatory cytokines [43]. - Matrine can attenuate S1P_1_ upregulation and astrogliosis in EAE [128]. - S1P_1_ activation can trigger ERK1/2 phosphorylation in mixed glial culture and astrocytes [129].
S1P**_2_**	- JTE013 can attenuate microglial activation, morphological transformation into ameboid cells, and proliferation in cerebral ischemia [87]. - JTE013 can attenuate proinflammatory responses of microglia and their NF-κB activation in cerebral ischemia [87]. - JTE013 or S1P_2_ knockdown can decrease mRNA expression levels of proinflammatory cytokines in LPS-stimulated BV2 microglia [87].	- N/A
S1P**_3_**	- CAY10444 can decrease microglial activation, morphological transformation into ameboid cells, and proliferation in cerebral ischemia [94]. - CAY10444 can attenuate proinflammatory responses of microglia and their NF-κB activation in cerebral ischemia [94]. - CAY10444 or S1P_3_ knockdown can decrease mRNA expression levels of proinflammatory cytokines in LPS-stimulated primary microglia [94].	- CAY10444 can decrease activation and proliferation of astrocytes in cerebral ischemia [94]. - S1P_3_ deletion can attenuate COX-2 upregulation in S1P-stimulated primary astrocytes [137]. - S1P_3_ deletion can reduce astrogliosis in Sandhoff disease [142]. - S1P_3_ knockdown in astrocytes can reduce mRNA expression of chemokines and proinflammatory cytokines [143]. - CAY10444 can reduce S1P-induced CXCL1 chemokine production from cultured astrocytes [91].
S1P**_4_**	- N/A	- N/A
S1P**_5_**	- N/A	- N/A

**Table 2:** LPA receptors in neuroglial activation and their neuroinflammatory responses. N/A: not available.

Receptors	Microglia	Astrocytes
LPA**_1_**	- AM095, AM152, or LPA_1_ knockdown can attenuate microglial activation, morphological transformation into ameboid cells, and proliferation in cerebral ischemia [160,162]. - AM095 can decrease mRNA expression levels of proinflammatory cytokines and attenuate microglial NF-κB activation in cerebral ischemia [162]. - LPA_1_ knockdown can reduce mRNA expression levels of proinflammatory cytokines in LPS-stimulated primary microglia [158,162]. - AM095 can attenuate microglial NLRP3 inflammasome activation in cerebral ischemia [161]. - AM966 can attenuate microglial activation in ICH [166]. - AM095 or LPA_1_ deletion can promote cell survival of oligodendrocytes that are exposed to conditioned medium of microglia treated with LPA [28]. - LPA_1_ knockdown can decrease microglial activation and proliferation in septic brain [158]. - Saikosaponin-d, which acts as an LPA_1_ inhibitor, can reduce apoptotic death of SH-SY5Y neurons cocultured with BV2 microglia and attenuate proinflammatory cytokines production from LPS-stimulated primary microglia [171].	- AM095 can reduce activation and proliferation of astrocytes in cerebral ischemia [162]. - LPA_1_ knockdown can reduce migration of IL-1β-stimulated astrocytes [196]. - Ki16425, AM966, or LPA_1_ knockdown can reduce ERK1/2 phosphorylation in LPA-stimulated C6 glioma [197]. - Ki16425 can attenuate astrocytic CXCL1 upregulation in neuropathic pain [198].
LPA**_2_**	- LPA_2_ deletion in microglia can promote cell survival of oligodendrocytes that are exposed to conditioned medium of microglia treated with LPA [173].	- N/A
LPA**_3_**	- DGPP can attenuate LPA-induced membrane ruffling of activated microglia [150]. - Ki16425 can attenuate ERK1/2 phosphorylation in LPA-stimulated microglia [149]. - Ki16425 can attenuate ROS production from LPA-stimulated EOC mouse microglial cells [176].	- Ki16425 can attenuate astrocytic CXCL1 upregulation in neuropathic pain [198].
LPA**_4_**	- N/A	- N/A
LPA**_5_**	- TCLPA5 can attenuate microglial activation, morphological transformation into ameboid cells, and proliferation in cerebral ischemia [179]. - TCLPA5 can decrease mRNA expression levels of proinflammatory inflammatory cytokines in cerebral ischemia [179]. - LPA_5_ knockdown can decrease mRNA expression levels of pro-inflammatory cytokines in LPS-stimulated BV2 microglia [179]. - TCLPA5 can reduce RAGE upregulation in activated microglia in cerebral ischemia [200]. - TCLPA5 can attenuate microglial NF-κB activation in cerebral ischemia [200]. - TCLPA5 can attenuate M1 polarization in LPA-stimulated primary microglia or BV2 microglia [178]. - AS2717638 can attenuate production of proinflammatory cytokines and chemokines from LPA-stimulated BV2 microglia [180].	- N/A
LPA**_6_**	- N/A	- N/A
